# Supplementary material for: Podoplanin Expression in Early-Stage Colorectal Cancer-Associated Fibroblasts and Its Utility as a Diagnostic Marker for Colorectal Lesions
Source: Cells. 2024 Oct 11;13(20):1682. doi: 10.3390/cells13201682 (PMC11506654; doi:10.3390/cells13201682)
Supplement: Supplementary file 1 [file cells-13-01682-s001.zip › Supplementary_Table_1.pdf]

Supplementary Table S1. Properties of pericryptal PDPN positivity and p53 aberrant expression for diagnosing non-neoplastic and neoplastic colorectal lesions at two candidate cutoff values.

	**Pericyptal PDPN positivity**		**p53 Aberrant expression**
	**3.72% Cutoff**	**10% Cutoff**	
Sensitivity (%)	93.7	87.4	47.2
Specificity (%)	91.2	97.1	100
PPV (%)	87.4	95.1	100
NPV (%)	95.7	92.2	74.6

PPV, positive predictive value; NPV, negative predictive value.
