# Supplementary figures and images for: Podoplanin Expression in Early-Stage Colorectal Cancer-Associated Fibroblasts and Its Utility as a Diagnostic Marker for Colorectal Lesions
Source: Cells. 2024 Oct 11;13(20):1682. doi: 10.3390/cells13201682 (PMC11506654; doi:10.3390/cells13201682)

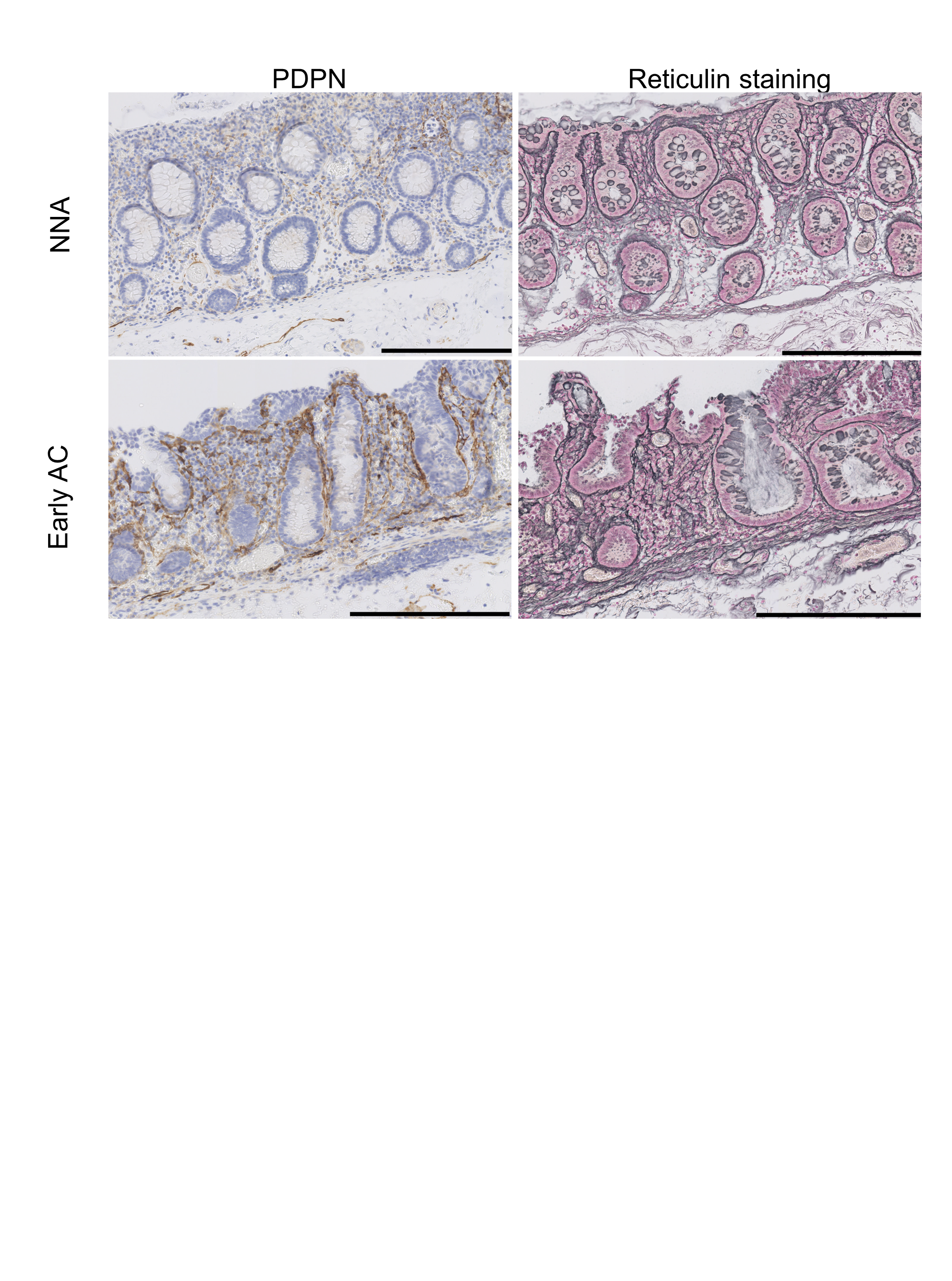

Supplement: Supplementary file 1 [file cells-13-01682-s001.zip › Supplementary_Figure_S1.TIF]

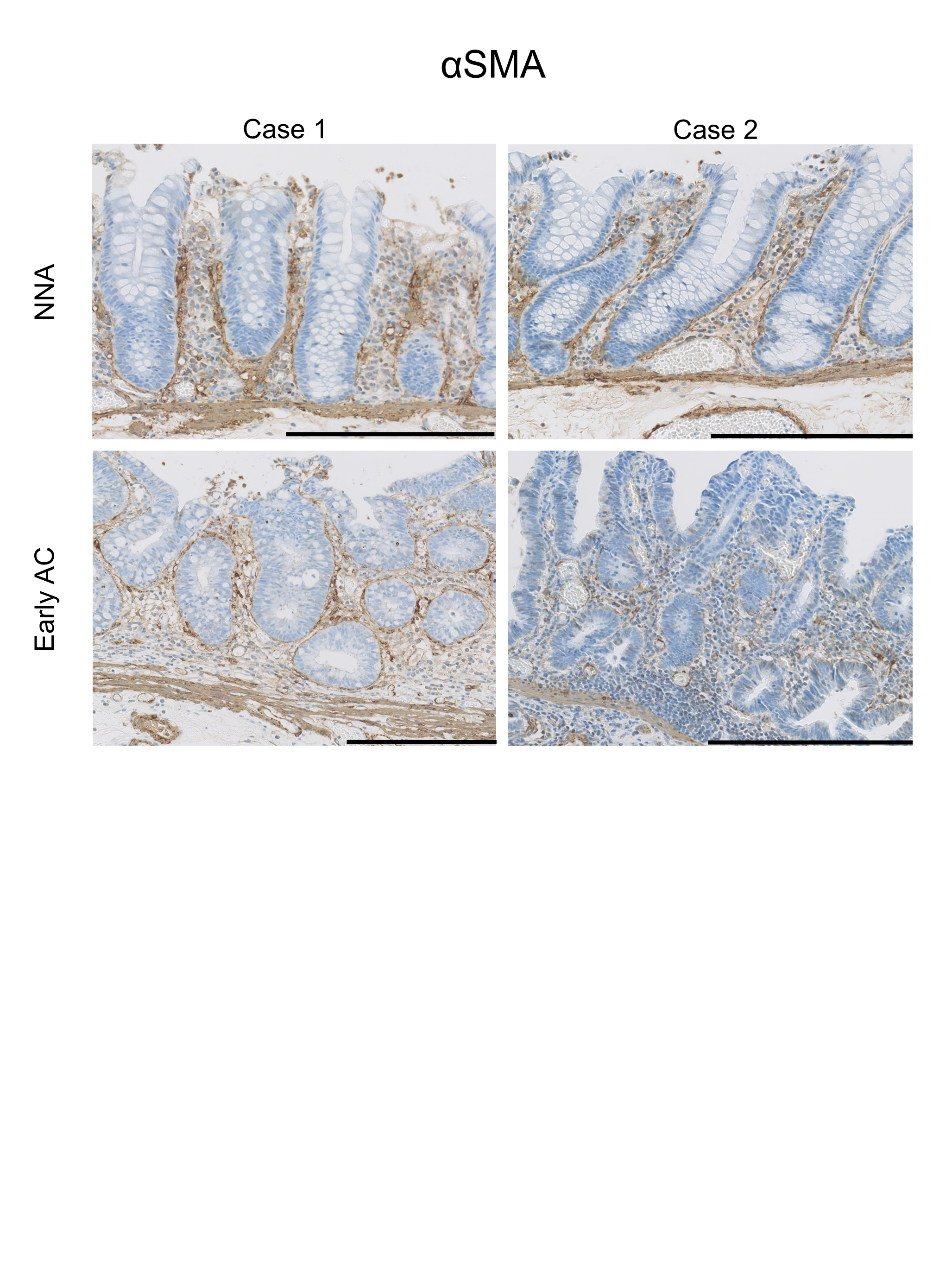

Supplement: Supplementary file 1 [file cells-13-01682-s001.zip › Supplementary_Figure_S2.TIF]

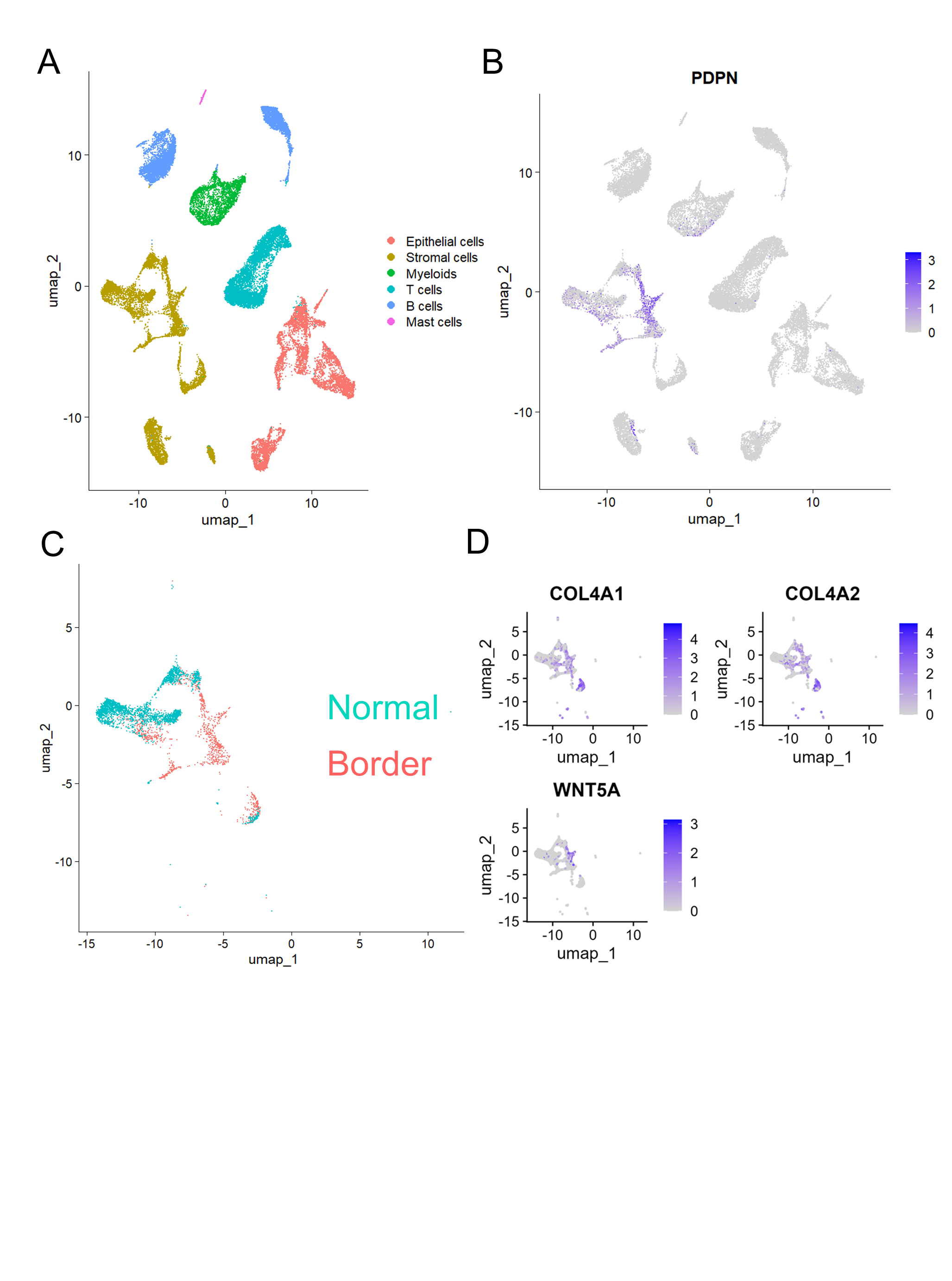

Supplement: Supplementary file 1 [file cells-13-01682-s001.zip › Supplementary_Figure_S3.TIF]

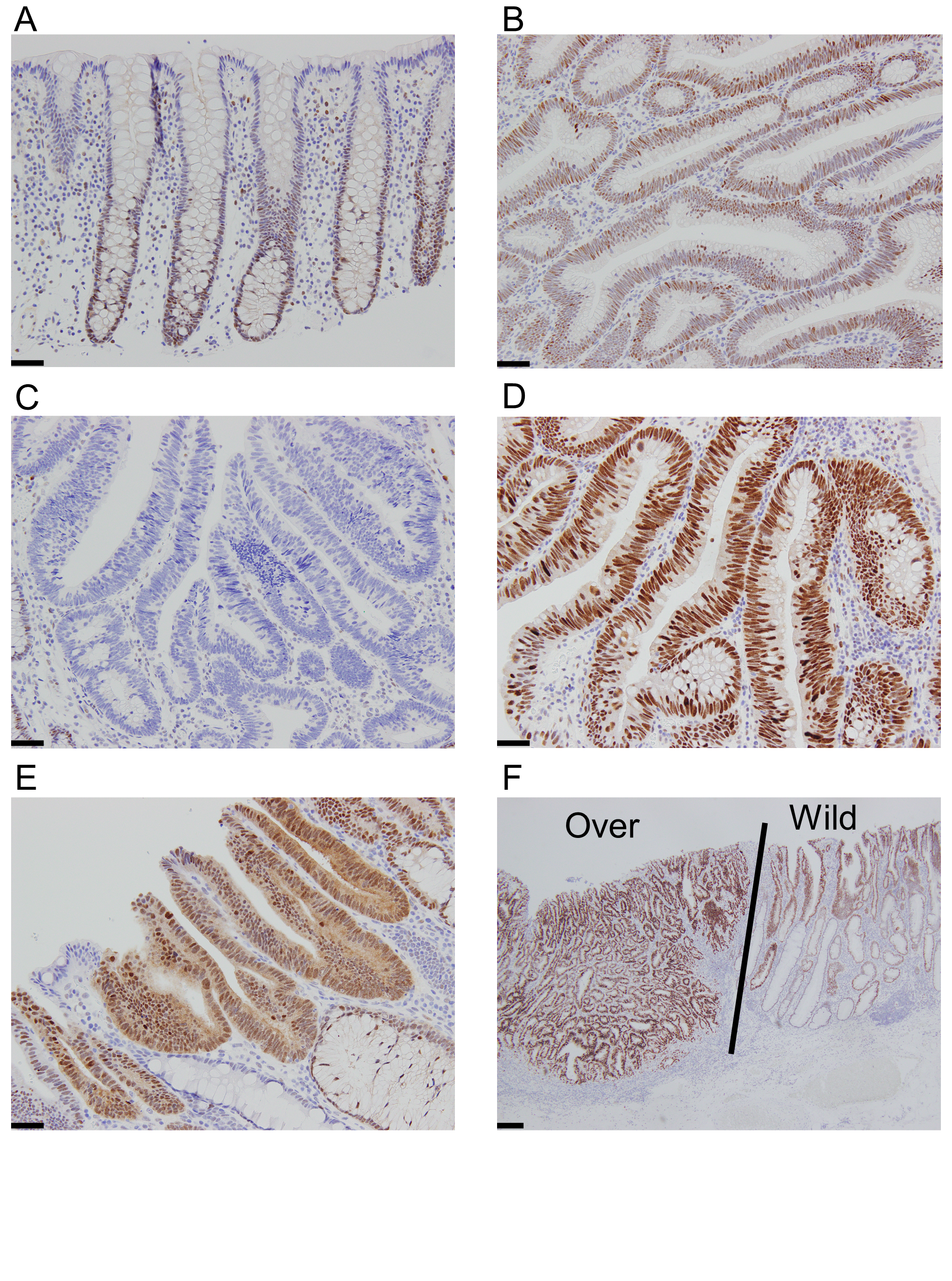

Supplement: Supplementary file 1 [file cells-13-01682-s001.zip › Supplementary_Figure_S4.TIF]
